# Nocturnal activity as a useful indicator of adaptability of dogs in an animal shelter and after subsequent adoption

**DOI:** 10.1038/s41598-023-46438-9

**Published:** 2023-11-03

**Authors:** Janneke Elisabeth van der Laan, Claudia Maureen Vinke, Saskia Stefanie Arndt

**Affiliations:** Animal Behaviour Group, Division of Animals in Science and Society, Department Population Health Sciences, Faculty of Veterinary Medicine, Utrecht University, P.O. Box 80166, 3584 CM Utrecht, The Netherlands

**Keywords:** Behavioural ecology, Behavioural methods

## Abstract

Dogs in shelters are faced with the challenge of adapting to a kennel after relinquishment and to a novel home after adoption. To measure adaptability of dogs, more feasible behavioural and physiological parameters need to be validated in different contexts. To evaluate nocturnal activity as an indicator of adaptability, we compared nocturnal activity, urinary cortisol:creatinine ratio (UCCR), and body weight changes of sheltered dogs the first period after intake in the shelter and after adoption. Nocturnal activity and UCCRs were significantly lower the first days after adoption than in the shelter. After adoption, nocturnal activity was significantly lower on night 2 than night 1, but not on night 3 and 4, suggesting a form of ‘rebound of resting’ during night 2 in the new home. UCCRs significantly decreased 7 days after adoption. Body weight decreased in the shelter but increased again after adoption. These findings suggest that overall, dogs rest better in a novel home than in a novel shelter but, in both contexts, some form of adaptation takes place. Nocturnal activity measured by an accelerometer differentiated well between shelter and home environments, and corresponded to UCCR responses, which supports usefulness of the method to monitor canine adaptability to novel environments.

## Introduction

Environmental stimuli and contexts abruptly change for dogs during the process of relinquishment to an animal shelter and subsequent adoption. To monitor the welfare of these dogs, it is of utmost relevance to evaluate if they are adapting to such new situations.

A shelter environment comprises many potential stressors (i.e., stress-evoking events and/or conditions) for dogs, such as high noise levels, unfamiliar scents, sudden separation from attachment figures, less exercise and unfamiliar dogs or people^1–4^. An inability to appropriately react/adapt to a novel environment and its (potential) stressors can lead to an impairment of animal welfare^5,6^. Previous studies detected stress responses in dogs after entering a shelter and kennel environment, reflected in physiological and behavioural changes. The hypothalamic–pituitary–adrenal (HPA) axis in the central nervous system is crucial in mediating such stress responses through the release of neurotransmitters and hormones. The steroid hormone cortisol, the primary glucocorticoid of dogs and many other species and the most reliable and widely used marker for HPA axis activity, was elevated for days up to even weeks after dogs entered a kennel environment^3,7–9^. Also, behavioural changes after kennel entry have been observed, including changes over time (days or weeks) in the kennel, which were described as markers of the stress response as they often concur with a change in other indicators of stress such as cortisol^7,10–14^. A physiological and behavioural response to a stressor can be an appropriate and functional adaptive response, but stress can become chronic if the individual dog fails to adapt to them over the longer term. This may exceed the animals’ adaptive capacity and thus, threaten its welfare state^6^. Chronic stress may even result in medical and behavioural problems in the longer term^15^.

Although less investigated, the transition into a new home after adoption presents a novel environment as well, also with similar challenges, such as new people and animals, and new routines. It has been described that dogs may behave differently in the period soon after adoption from a shelter then later on, with no or less problem behaviour display during the first period in the new home and a worsening of problem behaviour over time, which is referred to as the ‘honeymoon period’^16,17^. Previously, the difference in stress responses of dogs between a kennel environment and a novel home has mostly been evaluated by comparing responses in a kennel with responses relatively long after adoption. For instance, Stephen and Ledger^8^ found cortisol levels in the urine (urinary cortisol:creatinine ratio, UCCR) of dogs in shelters over a 31-day period to be higher than in a sample taken in the home environment 6 months after adoption. Comparably, Van der Laan and colleagues^18^ found UCCR levels of dogs to be higher in the shelter than 6 weeks after adoption. Behavioural changes after adoption have also been studied, mainly by comparing in-shelter behavioural evaluations with behaviour as reported by the new owner after adoption^19^, or comparing the behaviour of the dogs on certain time points (weeks) after adoption with an owner-reported questionnaire^17,20^. However, studies that compare responses of dogs during the transition from a shelter to a novel home the first days after adoption are scarce^21^. A good controlled study comparing transitions to a shelter environment with the transitions to a novel home environment makes it possible to elucidate the real impact of stressors typical for a shelter or whether it is merely the novelty of the environment that makes it harder for dogs to adapt to a shelter environment. Also, identifying individual dogs who are not adapting well to a shelter or novel home environment can help shelters and owners to provide extra support for these dogs, e.g., by removing stressors where possible, choosing another (quieter) kennel, providing hiding opportunities, provide more human contact, mental enrichment, etc.

Previous research found resting patterns to be disturbed in stressful environments such as in animal shelters. In general, domestic urban dogs have sleep cycles of on average 21 min during the night, with 16 min asleep and 5 min awake^22^. The percentage asleep and number of sleeping bouts can differ between types of environments, such as a restricted or unrestricted context (fenced or unfenced property). Dogs in an animal shelter spent 45% of their time asleep over a period of 24 h, and 72% of the night-time (17:00–08:00)^23^. Another study found sheltered dogs to lie with their head down for around 87–90% during the night (19:00–07:00)^24^. Elderly dogs (8–13 years) did not sleep between 14:00 and 16:00 on the first day in the shelter, but on day 6 they spent 43% of this timeslot asleep^25^. Discrepancies between studies can be due to differences in scoring patterns of sleeping/lying head down behaviour. Generally, behavioural studies are labour-intensive, which argues for using an objective non-invasive measure of inactivity-activity or behavioural sleep, such as an accelerometer^26^.

Activity patterns for dogs in shelters are known to be significantly different from dogs in homes. For example, during the night, which is the least active period for both sheltered and owned dogs, sheltered dogs had higher average activity levels than owned dogs^27^. Gunter and colleagues^21^ found dogs to have longer bouts of uninterrupted rest and lower UCCR levels during a 1 or 2-day fostering period in a home environment than pre- and post-fostering in the shelter. Hoffman and colleagues^27^ and Gunter and colleagues^21^ therefore suggested that a shelter environment might inhibit dogs from resting and that dogs therefore may experience sleep deficits in shelters. In humans, sleep deprivation is associated with numerous physical and mental health problems^28,29^ and might therefore be a welfare problem in itself. In addition, recovery of resting patterns seems a useful indicator of adaptability to novel environment in dogs^14^, as has also been suggested in farm animals decades ago^30^.

Previously, we evaluated nocturnal activity in sheltered dogs during the first period in a shelter^14^ and found a significant decrease in activity from the first nights to later nights in the shelter. Post-adoption evaluations, immediately after adoption, were not included and is the focus of the present study. The present study has a three-fold aim. First, we compared nocturnal activity during the first period in the shelter on different days of stay to the nocturnal activity during the first period after adoption in the same dogs. Hereby, we aimed to make better claims about the stressful effect on dogs of particular stressors in a shelter or merely novelty changes in the shelter environment. Second, we measured changes in nocturnal activity over the first nights after adoption with the aim to evaluate whether dogs adapted to their new home after adoption. And third, to further validate nocturnal activity measured by accelerometers as an indicator of adaptability, we also evaluated the physiological and physical response of the dogs by comparing UCCR levels and changes in body weight in the first period in the shelter to the same measures in the first period in the new home.

Based on previous research, we expected significantly lower overall nocturnal activity after adoption than in the shelter. However, we also expected some form of habituation to the novel home environment after the shelter period, and therefore a decrease in nocturnal activity from the first to later nights. We expected UCCR levels to follow the same pattern as nocturnal activity measures, as showed in our last study validating nocturnal activity as a measure of adaptability^14^.

## Methods

### Subjects

Thirty-one dogs that entered the largest animal shelter in the Netherlands (Animal Shelter DOA) between October 2018 and August 2019 were included in the study. For demographics per dog see Supplementary Table S1. These dogs were a subset of the shelter dog group described in two of our previous studies^14,31^, which comprised the dogs of this group that were adopted by owners who agreed to participate in the study. For further inclusion criteria, see Van der Laan and colleagues^14,31^. We included 9 female (3 intact, 4 neutered, 2 unknown) and 22 male (16 intact, 6 neutered) individuals of various breeds and ages (mean 3.6 years, range 1–13 years). Dogs were either strays (*n* = 12) or relinquished by their owners (*n* = 19). Of the relinquished dogs, 7 were known to have been kennelled before in boarding kennels, kennel in backyard etc., 4 were kennelled for the first time and 8 had an unknown kennel history. All stray dogs had an unknown kennel history. Sheltered dogs were assigned to weight classes < 10 kg (*n* = 10), 10–20 kg (*n* = 7), > 20–30 kg (*n* = 7) or > 30 kg (*n* = 7), and age classes 1–4 years (*n* = 24), 5–8 (*n* = 4) and 9–13 (*n* = 3).

A control group of 21 pet dogs were also monitored in their own homes, during their normal routine. This group was the same control group as described in Van der Laan and colleagues^14,31^ and had the following characteristics: mean age 3.7 years (range 1–11 years), 8 females (7 neutered) and 13 males (11 neutered).

### Housing and visiting procedure

Dogs were individually housed in kennels with an inside glass-fronted and outside bar-fronted enclosure (both ~ 5 m2), separated by a hatch. The kennels were accessible by staff and volunteers between 8:00 and 17:00 to care for the dogs. Kennels were cleaned once every day between 8:00 and 13:00. Most dogs were fed dry kibble 2 times a day and some 3 times when needed. In the afternoon, food enrichment was provided for the dogs, such as a stuffed Kong® or bones. Food intake of the last meal of the dogs was evaluated visually every time before their next meal by one of the researchers and was categorised as 0 (none or almost none eaten), 1 (some, about half, eaten), 2 (all eaten). After two weeks in the shelter, dogs were divided in low food intake dogs (mean of all categorised numbers < 1, *n* = 6) or medium to high food intake dogs (mean of all categorised numbers ≥ 1, *n* = 25). Dogs were allowed out on a playing field once or twice a day (depending on individual dog needs, available space and personnel) for 30 min up to 90 min, with other dogs if possible. After full vaccination, which depended on previous known vaccinations of the dogs based on information in the dog’s passports, dogs were allowed to walk with volunteers every day or every other day in the area around the shelter for 20–45 min each time. Before dogs were fully vaccinated, volunteers spent similar time periods with the dogs in the shelter area.

The dogs in this study were adopted by their new owners after 15 days (min) up to 455 days (max) in the shelter (mean = 76 days). Dogs stayed either a relatively short (< 6 weeks, *n* = 12), medium (6–12 weeks, *n* = 12) or long (> 12 weeks, *n* = 7) time in the shelter before they got adopted. The new owners of the dogs were completely informed during the adoption conversation from the shelter and agreed to participate voluntarily. The new owners signed an informed consent and were provided with a written training leaflet and a set of materials for collecting urine samples of their dogs. One of the researchers visited the new owners 2 and 6 weeks after adoption to recollect the samples that the owners gathered. Owners were instructed to follow their routine as they would normally do with a new dog.

The dogs of the control group followed their normal routines with their owners in their own homes. Owners of control dogs in homes participated voluntarily, signed an informed consent, and followed their normal routine with their dog during the measurement period. They were also trained to collect urine from their dog by an instruction form and explanatory video.

### Nocturnal activity

Nocturnal activity was measured using a tri-axial accelerometer, the Actigraph® (Actigraph Corp, USA). Protective hard-plastic cases fitted special for the Actigraph® were 3D printed at our university. After intake in the shelter, the dogs were fitted with a collar with 4–5 fingers space between collar and neck. The Actigraph® in its protective case was fixed to the collar with duct-tape. Dogs wore the accelerometer for the full first 14 days in the shelter^14^, but only the data collection of night 1–4 was used for this study. After adoption, dogs wore the same accelerometer and same collar (or a similar collar with the same size in a few cases where their previous collar was not available), which was fitted prior to or during the adoption conversation with the new owners. New owners were asked to leave the collar on for at least the first three days and nights; some owners decided to leave it on also on the 4th night which provided us with an extra night of data. Dogs in the control group were visited by one of the researchers to fit a collar with accelerometer. Control dogs wore the collar for at least 3 consecutive nights.

Actigraph® data was processed and analysed with the accompanying software, ActiLife®. We used 15 s epochs as a standard, as in our previous studies^14,18^ and to allow for a more detailed analysis than when epochs would be set at for example 60 s. In ActiLife®, the following activity measures were calculated for the nocturnal time frame from 0:00 to 4:00: (1) Vector Magnitude Counts per minute (*VMCpm)*, which is the overall summed (Vector Magnitude) counts divided by the total duration of analysis in minutes, as a measure of total activity (frequency and intensity); (2) percentage of time spent active (*% active*), which is all summed 15 s epochs during which activity was determined (> 0 counts); (3) number of inactive bouts *(# inactive*), which were the number of bouts that no activity (0 counts) was determined, as a measure of sleep fragmentation and restlessness; and (4) number of inactive bouts > 15 min (*# inactive* > *15 min*), which only counted the number of inactive bouts that took longer than 15 min, to evaluate how often the dogs had the opportunity to fulfil a sleep cycle (on average 16 min^22^), see Table 1.

**Table 1:** ActiGraph® activity measures.

ActiLife® measures	Abbreviation	Description	Indicative of:
Vector Magnitude Counts per minute	*VMCpm*	The overall counts divided by the total duration of analysis in minutes	Total activity, both frequency and intensity
Percentage of time spent active	*% active*	Summed all 15 s epochs during which activity was determined	Total duration of activity
Number of inactive bouts	*# inactive*	Number of bouts in which no activity was determined	Sleep fragmentation and restlessness
Number of inactive bouts that took longer than 15 min	*# inactive* > *15 min*	Number of bouts in which no activity was determined, for longer than 15 min	How often dogs could fulfil a sleep cycle (of on average 16 min^22^)

As processed and analysed by the ActiLife® software. The four used measures are described, including abbreviation used in the text and a description of what these measures are indicative of regarding activity and sleep/rest of the dogs.

### Urinary cortisol:creatinine ratio (UCCR)

Urine samples of the dogs were taken in the morning on day 1 (after intake/adoption), 2, 3, 7 and 12, both after intake at the shelter (by the researchers) and after adoption (by the new owners). Since we have previously found that UCCRs 6 weeks after adoption were similar to UCCRs of dogs in homes that had not been in the shelter^18^, a 6-week sample was also collected both in the shelter and after adoption. For the control group, two morning urine samples (12 days apart from each other) were taken by the owners.

Urine sampling took place as follows. Dogs in the shelter were taken out of their kennels between 7:30 and 11:30 (median 8:45) on measurement days by one of the researchers. After adoption, dogs were taken out by their new owners between 6:00 and 12:00 (median 8:15) conform their usual routine. Urine of control dogs in homes was collected between 6:20 and 11:00 (median 8:30). Naturally voided morning urine was captured mid-stream with a ladle and transferred immediately with a disposable pipette to a vial (polypropylene, 5 mL, 75 × 13 mm, Sarstedt AG & Co). If the dogs in the shelter were not naturally urinating outside of their kennel they had probably urinated in their kennel before. Urine on the floor in the in- or outside kennel was then used, as in a previous study, we found no difference in UCCR when comparing these collection methods in our pilot study, even if the urine was disposed few hours earlier^18^. Samples were immediately stored in a − 20 °C freezer in the shelter, or at − 10 to − 20 °C in their owner’s freezer and transferred to a − 80 °C freezer within 32 days. Urine samples were analysed by the University Veterinary Diagnostic Laboratory of the Faculty of Veterinary Medicine at the Utrecht University, the Netherlands, as described in Van der Laan and colleagues^18^.

### Proportional body weight changes

As body weight loss can be stress-related in dogs^14,18,32^, dogs were weighed on a scale in the shelter (AllScales® Europe) or in the dog’s new home (PCE Instruments, PCE-PB 150N) on days 1, 12 and after 6 weeks. To calculate proportional body weight change in the shelter, body weight at shelter intake was used as a reference weight (= 100%) to compare body weight after 2 and 6 weeks in the shelter (new weight / reference weight). Similarly, to calculate post adoption proportional body weight change, body weight at the moment of adoption was used as a reference weight (= 100%) to compare body weight after 2 and 6 weeks after adoption.

### Statistical analysis

Data were stored and cleaned in Microsoft Excel® files (Microsoft Corporation). Statistical software program RStudio (version 1.0.136—©RStudio, Inc.) was used to perform linear mixed model analysis with the package ‘Nlme’^33^ and visual normality distribution was evaluated with the packages ‘ggplot2’ and ‘ggpubr’. Graphs were created in Graphpad Prism (version 8.3.0—©GraphPad Software, LLC).

Outcome variables were evaluated for normality by performing Shapiro–Wilk tests and visual inspection of boxplots and quantile–quantile plots of the data. The data of UCCR, nocturnal *VMCpm*, *% active*, and *# inactive* were right-skewed and therefore (natural) log-transformed before *t*-tests and inclusion in mixed models and back transformed for interpretation. Back-transformed (exp) log model values resulted in ratios, with a ratio < 1 meaning a lower value and > 1 a higher value than the reference mean. Alpha level was set at p < 0.05. For mixed models, 95% confidence intervals (CI) ranges < 1 or > 1 were considered significant. For mixed models with non-transformed data (*# inactive* > *15 min* and proportional body weight change), 95% confidence intervals with ranges < 0 or > 0 were considered significant.

Two linear mixed effects models were fit per measure for all outcome measures for nocturnal activity (*VMCpm*, *% active*, *# inactive, # inactive* > *15 min)* and UCCR: one model that included both in-shelter and post-adoption data for comparison between the two contexts and effect of potential explanatory factors, and one model with only post-adoption data to evaluate changes over time after adoption and effect of potential explanatory factors. For proportional change in body weight, one in-shelter model and one post-adoption model were fit, both including potential explanatory factors. All models included a fixed effect for ‘day’ (UCCR/body weight) or ‘night’ (nocturnal activity) and a random effect for ‘dog ID’ (individual identity). For the in-shelter and post-adoption models, we also included a fixed interaction effect for ‘day’ and ‘environment’ (in-shelter/post-adoption), as this was our main interest, although the factor environment was mostly best included as a main effect rather than an interaction. With the starting models, which included these fixed effects and all the explanatory variables described below, explanatory variables were dropped based on a backward selection approach, using the Akaike information criterion (AIC) to determine the best model fit with Maximum Likelihood estimation. The explanatory variables that were included in the start models to evaluate their effect on parameter variabilities were: ‘sex’ (female/male), ‘age class’ (1–4/5–8/9–12 years), ‘weight class’ (< 10 kg/10–20 kg/ > 20–30 kg/ > 30 kg), ‘relinquishment type’ (type of admission to the shelter: stray/relinquished), ‘kennel history’ (whether dogs had a known history in a kennel environment: yes/no/unknown), ‘time spent in the shelter’ (short: < 6 weeks/medium: 6–12 weeks/long: > 12 weeks), and ‘food intake’ (low/medium or high). When visual inspection of boxplot graphs revealed potential interactions between ‘day’, ‘night’ or ‘environment’ and one of the explanatory variables, this interaction was also added in the start model.

Final models were tested with various correlational and variance structures (with autoregressive model of the order 1 (AR1) correlation structure or weights) to test the best fit. Restricted Maximum Likelihood estimation was used for the final model. Models were evaluated by visual inspection of the residuals (normality and constant variance).

All final mixed model results, including the significance values (*p*-value), are given in the supplementary tables mentioned in the text.

For the control dogs in homes, the mean of the two UCCR measurements (averaged control dogs samples of day 1 and 12), and the mean of the three consecutive nights of nocturnal activity data (night 1–3), were calculated per dog to determine one outcome per parameter for control dogs in homes to compare with the in-shelter and post-adoption shelter dog samples, using an independent samples one-sided *t*-test (expectation = ‘greater’) on the log-transformed data. For nocturnal activity parameters, these outcomes were compared to shelter dog night 3 after adoption (*n* = 30), as night 4 after adoption comprised fewer data points (*n* = 20), also using an independent samples one-sided *t*-test (expectation = ‘greater’) on the log-transformed data.

### Statement of ethical approval

Methods in this study were performed in accordance with relevant guidelines and regulations. All dog owners agreed and volunteered to participate in this study and signed informed consent. All experimental protocols were approved by the institutional committee Utrecht Animal Welfare Body of Utrecht University, The Netherlands.

## Results

### Best model fits and effect of day/night in-shelter and post-adoption

**Figure 1:**
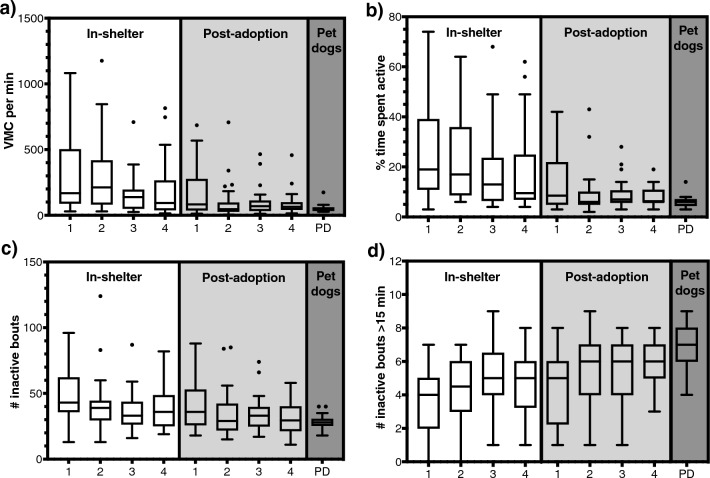
Box and whisker (Tukey) plots with median and outliers (dots) over time in the shelter and after adoption for nocturnal activity parameters: (**a**) *VMCpm*, (**b**) *% active*, (**c**) *# inactive* and (**d**) *# inactive* > *15 min*. On day (*x*-axis**) **1, 2, 3 and 4 in the shelter (white area) and after adoption (light grey area), and the averaged data of 2 nights in a control group of pet dogs (PD) in their own homes (dark grey), during 4-h measurement periods from 0:00 to 4:00 AM.

For best model fits for all parameters, including best fitting variance and correlational structures, see Table 2. Night/day/week explained variable variances for all parameters, see Fig. 1 for nocturnal activity parameter results over time in the shelter and after adoption, and Supplementary Tables S2–S10 for all final mixed model outcomes.

**Table 2:** Best model fits for nocturnal activity parameters, UCCR and proportional body weight, including correlational and variance structures.

		Model with in-shelter and post-adoption combination data		Model with only post-adoption data	
Parameter		Fixed factors	Structures	Fixed factors	Structures
Nocturnal Activity	*VMCpm*	Night*environment Environment*relinquishment type	Variance for environment	Night	CAR1 + Power variance for night
	*% active*	Night*environment Environment*kennel history	Variance for environment	Night	CAR1 + Power variance for night
	*# inactive*	Night*environment Time spent in the shelter Environment*kennel history Environment*relinquishment type	None	Night	None
	*# inactive* > *15 min*	Night*environment Time spent in the shelter Environment*kennel history Environment*relinquishment type	None	Night	None
UCCR		Day*environment Weight class Environment*kennel history	Variance for environment	Day Weight class	None
Parameter		Model with only in-shelter data		Model with only post-adoption data	
Body weight		Week*weight class Relinquishment type Food intake	None	Week Weight class	None

A ‘*’ indicates an interaction between factors. All in-shelter and post-adoption combination models included a night/day*environment interaction to compare nights in-shelter with post-adoption. A variance structure for environment allowed for different residual variances for in-shelter versus post-adoption. A power variance function structure for night modelled a relationship between nights and the variance. A CAR1 is a continuous autocorrelation structure of order 1.

### Nocturnal activity of the sheltered dogs: influence of the factors relinquishment type, kennel history and time spent in the shelter

**Figure 2:**
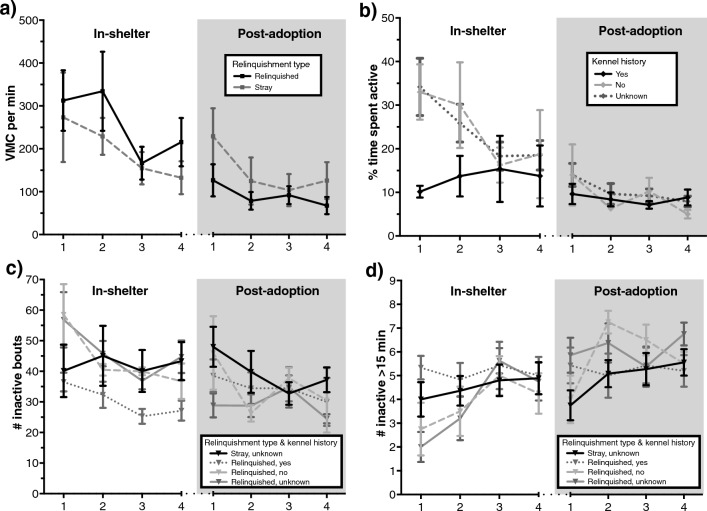
Mean ± SEM over time in the shelter and after adoption for nocturnal activity parameters: (**a**) *VMCpm*, (**b**) *% active*, (**c**) *# inactive* and (**d**) *# inactive* > *15 min* over time in the shelter and after adoption, categorized for relinquishment type and kennel history. On day (*x*-axis) 1, 2, 3 and 4 in the shelter (white area) and after adoption (light grey area), during 4-h measurement periods from 0:00 to 4:00 AM.

Not all nights contained accelerometer data of all sheltered dogs, due to missing data on some days caused either by material failure or shortage of accelerometers. The samples gathered of the total 31 sheltered dogs are: in-shelter night 1 *n* = 23, night 2 *n* = 26, night 3 *n* = 29, night 4 *n* = 28; after adoption night 1 *n* = 28, night 2 *n* = 31, night 3 *n* = 30, night 4 *n* = 20. See Fig. 2 for the influence of factors on nocturnal activity parameters.

#### VMCpm

VMCpm indicating activity, were significantly lower during all nights (1–4) after adoption than during all nights (1–4) in the shelter for relinquished dogs (Fig. 2a, see Supplementary Table S2 for mixed model results). In the research population, stray dogs had significantly lower levels on night 2 after adoption compared to night 2 in the shelter, but other nights did not significantly differ. Although an interaction between environment and relinquishment type was included in the best fitting model for *VMCpm*, and stray dogs had higher *VMCpm* than relinquished dogs after adoption, no significant differences were found based on the 95% CI. After adoption (Supplementary Table S3), *VMCpm* was significantly lower on night 2 compared to night 1, and lower but not significantly on night 3 and 4 compared to night 1.

#### % active

% active was significantly lower during all nights (1–4) after adoption than during all nights (1–4) in the shelter for dogs that had no kennel history or unknown kennel history (Fig. 2b, Supplementary Table S4). Dogs with a known kennel history only were a lower *% active* during night 2 after adoption compared to night 2 in the shelter. Overall, in the shelter, dogs with a known kennel history were a significantly lower *% active* than dogs with no kennel history and dogs with unknown history, and this difference disappeared after adoption. After adoption (Supplementary Table S3), the *% active* was significantly lower on night 2 compared to night 1, and lower but not significant on night 3 and 4, conform *VMCpm* results.

#### # inactive

# inactive, indicating fragmented sleep or restlessness, was significantly lower during all nights (1–4) after adoption than in the shelter for relinquished dogs with an unknown kennel history, and lower during night 1–2 after adoption than in the shelter for relinquished dogs with no kennel history (Fig. 2c, Supplementary Table S5). For stray dogs, no significant difference in *# inactive* for all days in the shelter compared to after adoption appeared. Dogs with a known kennel history expressed a lower *# inactive* in the shelter than dogs with an unknown history or no history. Time spent in the shelter was included for best model fit but did not have a significant effect on the *# inactive* based on a 95% CI. After adoption (Supplementary Table S3), the *# inactive* was significantly lower during night 2 compared to night 1, and lower but not significantly during night 3 and 4, conforming *VMCpm* and *% active* results. Also, after adoption, strays expressed a higher *# inactive* than relinquished dogs.

#### # inactive > 15 min

# inactive > 15 min was significantly higher during all nights (1–4) after adoption than in the shelter for relinquished dogs with no kennel history or an unknown kennel history (Fig. 2d, Supplementary Table S6). However, for stray dogs with an unknown kennel history and relinquished dogs with a known kennel history no significant difference between nights 1–4 in the shelter compared to after adoption appeared. After adoption, overall, dogs with a known kennel history expressed a lower *# inactive* > *15 min* than dogs with an unknown kennel history, and stray dogs had lower *# inactive* > *15 min* than relinquished dogs. In the shelter, there was no overall difference between kennel histories and relinquishment types. In addition, with shelter and post adoption data grouped, dogs that stayed for a medium period (6–12 weeks) in the shelter expressed a lower *# inactive* > *15 min* than short stay dogs (< 6 weeks) and long stay dogs (> 12 weeks). After adoption (Supplementary Table S3), the *# inactive* > *15 min* was significantly higher during night 2 compared to night 1, and higher but not significantly during night 3 and 4, conform results of all other nocturnal activity parameters.

### Nocturnal activity: comparison with control group of dogs in homes

For comparisons between the nocturnal activity of the control group and shelter dog group after adoption, night 3 after adoption (which contained data of 30 dogs) was compared to the averaged data of 3 nights for control dogs in their own homes with normal routine (see Fig. 1 for control dog nocturnal activity results in comparison with results in the shelter and after adoption). All nocturnal activity parameters measured during night 3 after adoption were significantly different from those of control dogs, with *VMCpm*, *% active* and *# inactive* being higher and *# inactive* > *15 min* being lower for the post-adoption group (independent samples one-sided *t*-tests, respectively: sample estimated mean difference [ratio] (SEMDR) = 1.41, 95% CI = 1.04 − ∞, t[46] = 1.92, *p* = 0.030; SEMDR = 1.35, 95% CI = 1.11 − ∞, t[48] = 2.53, *p* = 0.007; SEMDR = 1.17, 95% CI = 1.02 − ∞, t[48] = 1.92, *p* = 0.031; SEMDR = -1.10, 95% CI = -∞ − -0.32, t[48] = -2.35, *p* = 0.011).

### Urinary cortisol/creatinine ratio (UCCR)

**Figure 3:**
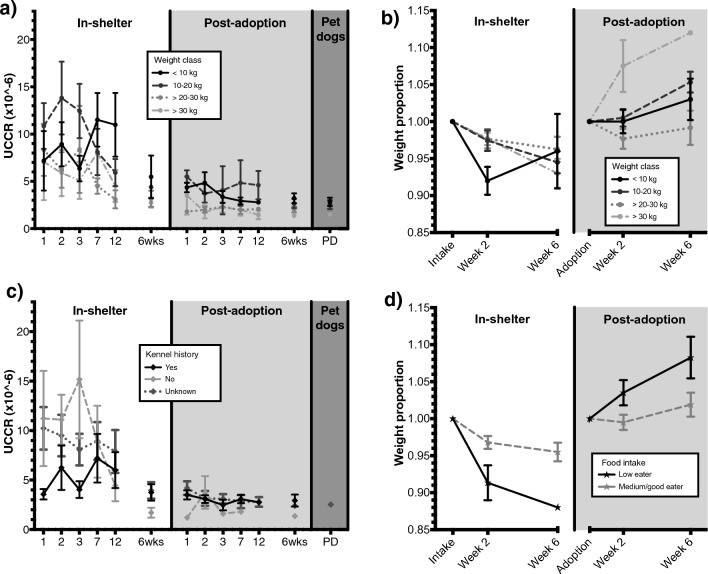
Mean ± SEM over time in the shelter and after adoption for urinary cortisol: creatinine ratio (UCCR, **a** & **c**) and proportional weight change (**b** & **d**) over time in the shelter and after adoption, categorized for weight class, kennel history or food intake. For UCCR on day (*x*-axis) 1, 2, 3, 7, 12 and 6 weeks in the shelter (white area), after adoption (light grey area), and the averaged UCCR data of 2 urine samples (12 days apart) in a control group of pet dogs (PD) in their own homes (dark grey area). For proportional weight change on intake/adoption, after 2 and 6 weeks in the shelter (white area) and after adoption (light grey area).

Not all urine samples of the sheltered dogs could be gathered on all measurement days by the researchers or owners. The samples gathered of the total 31 sheltered dogs are in: shelter day 1 *n* = 27, day 2 *n* = 28, day 3 *n* = 29, day 7 *n* = 29, day 12 *n* = 28, 6 weeks *n* = 18; after adoption day 1 *n* = 17, day 2 *n* = 17, day 3 *n* = 17, day 7 *n* = 17, day 12 *n* = 16, 6 weeks *n* = 21. See Fig. 3a,c for the influence of factors on UCCR.

In the shelter, UCCR levels of dogs were significantly higher than after adoption on all days (Fig. 3a,c), except the dogs with a known kennel history after 6 weeks in the shelter compared to after 6 weeks after adoption (Fig. 3c, mixed model results in Supplementary Table S7). In the shelter, UCCRs of dogs with a known kennel history were significantly lower than the UCCRs of dogs that had no kennel history or an unknown kennel history, but this difference was not found after adoption. In a model with only post-adoption data (mixed model results in Supplementary Table S8), UCCRs were significantly lower on day 7, 12 and 6 weeks after adoption compared to day 1 in the new home.

Overall, when data in shelter and after adoption were grouped, UCCRs of smaller dogs (< 10 kg and 10–20 kg) were higher than those of larger dogs (20–30 kg and > 30 kg, Fig. 3a). In the UCCR mixed model with data after adoption, weight class also significantly explained UCCR variability with the same effect.

For comparisons between the UCCR of control group dogs and sheltered dogs after adoption, day 3 and day 12 after adoption (for both *n* = 7) were compared to the averaged data of two UCCR outcomes (12 days apart) for control dogs in homes without novelty experiences (*n* = 21). No significant differences were found between UCCR at day 3 and 12 after adoption compared to those of control dogs (independent samples one-sided *t*-tests, day 3 vs averaged control dogs: estimated mean difference [ratio] = 1.12, 95% CI = 0.86—∞, t^33^ = 0.73, *p* = 0.235; day 12 vs averaged control dogs: estimated mean difference [ratio] = 1.12, 95% CI = 0.87—∞, t^34^ = 0.77, *p* = 0.224).

### Body weight proportional change

In the shelter, a significant decrease in proportional body weight from intake (100%) to week 2 (original values: mean = 95.7%, standard deviation (SD) = 4.9%) was found, and no significant difference between week 2 and week 6 (original values: mean = 95.0%, SD = 4.8%) in the shelter. See Fig. 3b,d for the influence of factors on proportional body weight change. Dogs that had a low food intake lost more weight in the shelter than dogs that had medium to high food intake (Fig. 3d). Although relinquishment type and an interaction between week and weight class were added for best model fit, no significant differences were found based on a 95% CI’s (mixed model results in Supplementary Table S9).

After adoption (mixed model results in Supplementary Table S10), no significant difference between proportional body weight at adoption (100%) and after 2 weeks (original values: mean = 100.1%, SD = 4.7%) was found. However, after 6 weeks (original values: mean = 102.9%, SD = 7.1%) the proportional body weight was significantly increased compared to the moment of adoption and after 2 weeks in the new home. Also, overall, after adoption larger dogs (> 30 kg) had significantly higher proportional weight gain than all smaller classes (Fig. 3b).

A paired *t*-test comparing absolute body weight in kilograms at intake and absolute body weight in kilograms 6 weeks after adoption showed no significant difference (sample estimated mean difference = 0.218, 95% CI = − 0.52–0.96, t[24] = 0.61, *p* = 0.55).

## Discussion

In this study, we compared nocturnal activity of dogs during the first period in an animal shelter to nocturnal activity of the same dogs during the first period after adoption, to elucidate the impact of stressors typical for shelters or whether it is merely the novelty of the environment that makes it harder for dogs to adapt to a shelter environment. Furthermore, we measured changes in nocturnal activity over the first nights after adoption to evaluate how dogs adapted to their new home. We also evaluated the physiological stress response by comparing UCCR levels in the first period in the shelter with UCCR levels during the first period in the new home, to further validate nocturnal activity measured by accelerometers as an indicator of adaptability.

The results show that nocturnal activity (vector magnitude counts per minute, percentage of time spent active and number of inactive bouts) and UCCR levels were significantly lower after adoption than in the shelter. After adoption, both nocturnal activity measures and UCCR levels decreased over time, indicating some form of adaptation. Three days after adoption, dogs were more active but UCCRs did not differ from control dogs in their own homes. Overall, this supports the conclusion that dogs show a lower stress response in a novel home than in a shelter, and therefore that dogs are better able to adapt to novel home environments than to novel shelter environments with particular stressors, but even in a new home dogs need time to adapt.

All stress-related parameters significantly decreased over time, both in the shelter and after adoption, showing some form of adaptation in the shelter and in the new home. However, responses were higher in the shelter than after adoption, and dogs returned to normal (i.e. control group) response levels quicker in a new home than in the shelter. This suggests that the shelter environment can be more challenging to dogs than a novel home environment, or in different words: it is not *just* the novelty of the shelter environment that causes the stress response. Two main components can explain this response. First, a shelter environment might have *more novel stimuli* than a home environment, such as more unfamiliar people, a shelter routine differs more from a home routine than home routines might differ from each other, or more (unfamiliar) noises. Another reason could be that there are more *specific or intense stressors* in a shelter environment that are less, or not, present in homes, such as loud noises (e.g., barking dogs), unfamiliar smells, having to urinate and defecate in own living space^34^, less exercise and attention, etc. For example, Adams and Johnson^22,35^ found dogs woke up due to barking dogs, and responded more to barking sounds than to other common urban sounds. Also, social isolation and spatial restriction can be stressors to dogs^9,36^. Importantly, these stressors may be different per shelter, as shelters vary greatly regarding the environment and management routines, depending on available resources for instance. In addition to those two potential explanations, or a combined effect, other factors might contribute as well. For example, social separation from previous attachment figures after entering a shelter can be quite stressful^3^, new owners may have more time to provide more emotional support than shelter staff can do, and dogs may spend less time alone especially during the first period in a new home. Both in the shelter and after adoption, it is important to early identify individuals that have a hard time adapting, and to provide them with strategies to support them during a transition to a shelter and/or to a new home. For example, providing more human interaction has shown to decrease stress levels in sheltered dogs^37–39^. Providing a quiet and comfortable resting and hiding place may also support these dogs in adapting, as shown in sheltered cats, where hiding boxes reduced acute stress at least 4 days earlier than cats without hiding opportunities^40,41^. Furthermore, these results highlight the relevance of considering alternative options to rehome dogs. For example, community rehoming can prevent dogs having to stay in the shelter, which is especially important for dogs unlikely to tolerate a shelter stay.

Prolonged disturbed or deprived resting or sleep is not only a useful measure to monitor adaptation, but can also have negative physical and mental consequences and can therefore be a welfare risk to dogs in a shelter. Dogs might get drowsy and irritable due to a lack of rest, like sleep deprived humans^42^, which can influence how they react to shelter staff or to new owners. Sleep deprived laboratory dogs showed behavioural changes, including increased inactivity (time spent laying and standing inactive), increased display of maintenance behaviours and decreased play and alert behaviours^43^. The relationship between sleep deprivation and the behaviour and mental state of sheltered dogs needs further study, as it may impact not only the welfare of dogs, but also the relationship with shelter staff and matching to potential adopters. After adoption, nocturnal activity, but not UCCR levels, showed that dogs were less active during the second night than the first night, but not always during the 3rd and 4th night. A potential explanation is that familiarisation with the whereabouts of the new home takes place during the first day and night, sufficient enough to allow for a rebound in sleep or resting behaviour the second night, compensating for missed rest or sleep in the shelter. After that, further familiarisation can take place.

Sleep deprivation is known to cause a rebound in REM sleep and total sleep time in dogs and other species^42–44^. To study the changes in macrostructure of sleep of dogs in shelter and after adoption, including (the recovery of) REM sleep, EEG recordings are necessary in future studies. Also, it is known that dogs might behave differently during the first period after adoption than after a longer period in the new home^17^. Stephen and Ledger^16^ described this as a ‘honeymoon period’, where dogs may behave differently during a period of habituation soon after adoption than after a longer period in their new home. Our data support this hypothesis as dogs need time to adapt to their new home, although there are differences between dogs (i.e., variability in responses) and the length of this period still needs more research. In this study, UCCRs returned to normal pet dog levels after 3 days in the new home, but nocturnal activity during the 3rd night was still deviant from pet dog nocturnal activity, suggesting that dogs needed more than 3 days to adapt.

Proportional body weight decreased significantly from shelter intake to week 2 in the shelter, but significantly increased from immediately after adoption to after living 6 weeks in the new home, back to body weight levels at intake in the shelter. These changes in body weight can be stress-related^32^ or related to other factors such as food amount or palatability, or fat/obese dogs losing weight as more than half of the general dog population is overweight^45^. However, body condition score had no effect on body weight loss or gain, so overweight dogs did not lose more weight in the shelter^14^. Dogs that had a low food intake lost more weight in the shelter, as they ate less. The explanation however can be two-fold: dogs could simply not like the food provided, or the dog may eat less due to stress.

Of the factors that were explored for their influence on the responses of the dogs, relinquishment type and kennel history had the largest effect on most parameters. Dogs with a known history in kennel environments were significantly less active during the night and had lower UCCRs than dogs that had no or an unknown kennel history, in the shelter but not after adoption. This implies that kennel experience might help dogs to adapt faster to a shelter environment. Stray dogs had lower stress responses in the shelter than relinquished dogs, concurrent with our previous findings reflected in hair cortisol^31^. This means that relinquished dogs with no kennel history are at highest risk of showing a longer or more pronounced stress response. Similar effects of previous kennel experience and of being stray or relinquished from home environments have been reported^9,11^. However, other studies did not find an effect of previous kennel experience^7^. The effects of habituating dogs to a kennel environment therefore need further investigation, but it can have positive practical applications to prepare dogs for a stay in a kennel environment later in life by prior positive training during for example the socialisation period.

In addition, we found a main effect of weight class on UCCR levels, both in the shelter and after adoption: smaller dogs (< 10 kg and 10–20 kg) had higher cortisol levels than larger dogs (20–30 kg and > 30 kg). Higher cortisol levels in smaller dogs have been described before (UCCR^14,21,46,47^; salivary^47,48^; and in hair^31^; but not in plasma^3^. Several potential explanations have been given, ranging from physical reasons like mass-specific metabolic rates^49^ and little creatinine production in smaller dogs due to muscle mass^50^, to mental reasons like higher stress vulnerability of small dogs in shelters, due to less socialisation and training in smaller breeds^51,52^. The results of this study pinpoint to the hypothesis that UCCR levels are higher in smaller dogs in general and therefore physical reasons, as we found an overall effect and no interaction with time.

One limitation of shelter studies is that the life history of the dogs at intake is generally unknown. Especially early life experiences can have a huge impact on the development of behaviour and individual abilities to adapt to novel environments and other environmental stressors^53^. Therefore, in welfare studies, information on the life history of the experimental subjects can add to understand individual adaptation profiles. Moreover, dogs that are taken in a shelter can be a biased population with behavioural problems affiliated to life history and might therefore not represent the average dog population. Another limitation of this study is that due to the handling of dogs during sample collection, dogs included in the study had more interaction with humans than generally dogs in the same shelter would, which might have influenced their stress response. For instance, dogs were taken out of their kennels more often for urine collection, fitting the collar with the accelerometer and weighing the dogs on a scale. Interactions with humans are known to decrease stress levels in sheltered dogs^37–39^ and might therefore have influenced our results, even though we aimed to minimize the effect by keeping handling moments to a minimum and did not interact with the dogs more than necessary for smooth sample collection.

To conclude, accelerometer-derived measurement of nocturnal activity differentiated well between a shelter and home situation, and this nocturnal activity corresponded to UCCR responses. Nocturnal activity can therefore be a useful non-invasive and practical parameter to monitor canine adaptability to a novel environment. Accelerometers are now available as relatively cheap equipment and can therefore be useful for shelters to monitor their dogs. As individual differences matter for monitoring welfare, it is important to identify dogs that have more difficulty adapting to the shelter environment, to provide them with additional and individualized support where possible. Dogs seem to rest and therefore adapt better in a novel home situation than in a novel shelter environment. A shelter environment poses more challenges to dogs, especially when they did not have previous experiences with kennelling. But even when being adopted in a novel home environment after a shelter period, dogs need some time to adapt. After all, a home isn’t built in a day.

### Supplementary Information

Supplementary Tables.

## Data Availability

The datasets generated during and/or analysed during the current study are available from the corresponding author on reasonable request.
