# Supplementary material for: Nocturnal activity as a useful indicator of adaptability of dogs in an animal shelter and after subsequent adoption
Source: Sci Rep. 2023 Nov 3;13:19014. doi: 10.1038/s41598-023-46438-9 (PMC10624664; doi:10.1038/s41598-023-46438-9)
Supplement: Supplementary file 1 — Supplementary Tables. [file 41598_2023_46438_MOESM1_ESM.docx]

**Supplementary information**

**Supplementary Table S1: Demographics per shelter dog and control pet dog.** Estimated breed group (Pollinger *et al.*, 2010^1^), age class (in years, Van der Laan *et al.*, 2021^2^), sex (female = f, male = m), neuter status (yes = y, no = n, unknown = ?), relinquishment type (relinquished = R, stray = S), body weight class (in kg), kennel history (yes = y, no = n, unknown = ?), and food intake label (low food intake or medium/high food intake during the first two weeks in the shelter).

| **Shelter/pet dog** | **Breed group** | **Age class** | **Sex** | **Neut. status** | **Relinq.**  **type** | **Kennel history** | **Body**  **weight** | **Eating during first two weeks** |
| --- | --- | --- | --- | --- | --- | --- | --- | --- |
| Shelter dogs | Ancient & spitz breeds | 1-4 yrs | f | ? | R | ? | 10-20 kg | Low |
|  | Ancient & spitz breeds | 1-4 yrs | m | n | S | ? | <10 kg | Low |
|  | Ancient & spitz breeds | 1-4 yrs | m | n | S | ? | 10-20 kg | Medium/good |
|  | Ancient & spitz breeds | 1-4 yrs | m | n | R | ? | <10 kg | Medium/good |
|  | Herding dogs | 1-4 yrs | f | y | R | ? | >30 kg | Low |
|  | Herding dogs | 1-4 yrs | f | y | R | n | >20-30 kg | Medium/good |
|  | Herding dogs | 1-4 yrs | m | n | R | ? | 10-20 kg | Low |
|  | Mastiff-like dogs | 1-4 yrs | f | y | R | y | <10 kg | Low |
|  | Mastiff-like dogs | 1-4 yrs | f | n | S | ? | >30 kg | Medium/good |
|  | Mastiff-like dogs | 1-4 yrs | m | n | R | y | >20-30 kg | Medium/good |
|  | Mastiff-like dogs | 1-4 yrs | m | n | S | ? | >30 kg | Medium/good |
|  | Mastiff-like dogs | 1-4 yrs | m | n | S | ? | >30 kg | Medium/good |
|  | Mastiff-like dogs | 1-4 yrs | m | n | R | ? | >20-30 kg | Medium/good |
|  | Mastiff-like dogs | 1-4 yrs | m | n | S | ? | 10-20 kg | Medium/good |
|  | Mastiff-like dogs | 1-4 yrs | m | n | S | ? | >30 kg | Medium/good |
|  | Mastiff-like dogs | 5-8 yrs | f | n | S | ? | >20-30 kg | Medium/good |
|  | Mastiff-like dogs | 5-8 yrs | m | n | S | ? | >20-30 kg | Medium/good |
|  | Mastiff-like dogs | 9-13 yrs | m | n | S | ? | 10-20 kg | Medium/good |
|  | Mixed (undefinable) | 5-8 yrs | m | y | R | n | >20-30 kg | Medium/good |
|  | Retrievers | 1-4 yrs | f | ? | S | ? | >20-30 kg | Medium/good |
|  | Retrievers | 1-4 yrs | m | y | R | ? | >30 kg | Medium/good |
|  | Retrievers | 1-4 yrs | m | n | R | y | <10 kg | Medium/good |
|  | Retrievers | 5-8 yrs | m | n | R | y | >30 kg | Medium/good |
|  | Scent hounds | 9-13 yrs | m | y | R | y | <10 kg | Medium/good |
|  | Small terriers | 1-4 yrs | f | n | R | n | 10-20 kg | Medium/good |
|  | Small terriers | 1-4 yrs | m | n | S | ? | <10 kg | Medium/good |
|  | Small terriers | 1-4 yrs | m | y | R | n | <10 kg | Medium/good |
|  | Small terriers | 1-4 yrs | m | y | R | ? | <10 kg | Medium/good |
|  | Small terriers | 9-13 yrs | m | n | R | y | <10 kg | Medium/good |
|  | Spaniels | 1-4 yrs | m | y | R | y | 10-20 kg | Medium/good |
|  | Toy dogs | 1-4 yrs | f | y | R | ? | <10 kg | Low |
| Pet dogs | Ancient & spitz breeds | 1-4 yrs | f | y |  |  | <10 kg |  |
|  | Ancient & spitz breeds | 1-4 yrs | f | n |  |  | 10-20 kg |  |
|  | Ancient & spitz breeds | 1-4 yrs | m | y |  |  | <10 kg |  |
|  | Ancient & spitz breeds | 9-13 yrs | m | n |  |  | >20-30 kg |  |
|  | Herding dogs | 1-4 yrs | m | y |  |  | 10-20 kg |  |
|  | Mastiff-like dogs | 1-4 yrs | f | y |  |  | >20-30 kg |  |
|  | Mastiff-like dogs | 1-4 yrs | f | y |  |  | >20-30 kg |  |
|  | Mastiff-like dogs | 1-4 yrs | m | y |  |  | >20-30 kg |  |
|  | Mastiff-like dogs | 1-4 yrs | m | y |  |  | 10-20 kg |  |
|  | Mastiff-like dogs | 5-8 yrs | m | y |  |  | >20-30 kg |  |
|  | Mastiff-like dogs | 5-8 yrs | m | y |  |  | >20-30 kg |  |
|  | Mastiff-like dogs | 5-8 yrs | m | y |  |  | >30 kg |  |
|  | Mixed (undefinable) | 1-4 yrs | f | y |  |  | 10-20 kg |  |
|  | Mixed (undefinable) | 1-4 yrs | m | y |  |  | 10-20 kg |  |
|  | Retrievers | 1-4 yrs | f | y |  |  | >20-30 kg |  |
|  | Retrievers | 5-8 yrs | f | y |  |  | >20-30 kg |  |
|  | Scent hounds | 1-4 yrs | m | n |  |  | <10 kg |  |
|  | Small terriers | 1-4 yrs | m | y |  |  | 10-20 kg |  |
|  | Small terriers | 1-4 yrs | m | y |  |  | <10 kg |  |
|  | Small terriers | 5-8 yrs | f | y |  |  | <10 kg |  |
|  | Toy dogs | 1-4 yrs | m | y |  |  | <10 kg |  |

^1^ Pollinger, J. P., *et al*. Genome-wide SNP and haplotype analyses reveal a rich history underlying dog domestication. *Nature* **464**, 898-902 (2010).

^2^ van der Laan, J. E., Vinke, C. M., van der Borg, J. A. M. & Arndt, S. S. Restless nights? Nocturnal activity as a useful indicator of adaptability of shelter housed dogs. *Appl. Anim. Behav. Sci.* **241**, 105377 (2021).

**Supplementary Table S2:** **Full model results of nocturnal activity accelerometer outputs: Vector Magnitude Counts per minute (*VMCpm*).** Estimated parameter values (EP) and 95% confidence intervals (CI) of *VMCpm* during the night (0:00-4:00 h) for night (after intake/adoption) and other factors that significantly explained *VMCpm* variability. Conditional F-testing revealed F, DF’s and significance of factors in the model.

|  | | | *VMCpm* | | | | | |
| --- | --- | --- | --- | --- | --- | --- | --- | --- |
| **Category** | | | Estimated | | Conditional F-test | | | |
|  |  |  | **EP** | **95% CI** | **F** | **NumDF** | **DenDF** | **Sign.** |
| Reference | Night 1, in the shelter, relinquished | | 198.96^1^ | 118.37 – 334.42 | 1431.54 | 1 | 175 | <.0001 |
| Environment | Post adoption versus in the shelter | Night 1 | 0.39^2^ | 0.23 – 0.66 | 9.39 | 4 | 175 | <.0001 |
|  |  | Night 2 | 0.26^2^ | 0.15 – 0.43 |  |  |  |  |
|  |  | Night 3 | 0.50^2^ | 0.31 – 0.80 |  |  |  |  |
|  |  | Night 4 | 0.52^2^ | 0.31 – 0.88 |  |  |  |  |
| Relinquishment type | Stray versus relinquished | In the shelter | 0.84^3^ | 0.46 – 1.52 | 3.65 | 2 | 175 | 0.0279 |
|  |  | Post adoption | 1.55^3^ | 0.95 – 2.54 |  |  |  |  |

^1^ Estimated mean in reference night, environment and relinquishment type.

^2^ Estimated ratio of mean of specified environment and mean of reference environment at the same night.

^3^ Estimated ratio of mean of specified relinquishment type and mean of reference relinquishment type in the same environment.

**Supplementary Table S3:** **Full model results of nocturnal activity accelerometer outputs after adoption.** Estimated parameter values (EP) and 95% confidence intervals (CI) of accelerometer outputs post adoption during the night (0:00-4:00 h) for night (after adoption) and other factors that significantly explained accelerometer outputs variability. Conditional F-testing revealed F, DF’s (NumDF = 3 and DenDF = 75 for all parameters) and significance of the factor ‘night’ for all parameters in the model.

|  | *Accelerometer outputs* | | | | | | | | | | |
| --- | --- | --- | --- | --- | --- | --- | --- | --- | --- | --- | --- |
|  | Night 1 | | Night 2 | | Night 3 | | Night 4 | | Conditional F-test | |  |
| **Output** | **EP** | **95% CI** | **EP** | **95% CI** | **EP** | **95% CI** | **EP** | **95% CI** | **F** | **Sign.** |  |
| *VMCpm* | 89.66^1^ | 60.64 – 132.59 | 0.62^2^ | 0.46 – 0.83 | 0.77^2^ | 0.55 – 1.08 | 0.76^2^ | 0.52 – 1.11 | 3.99 | 0.0108 |  |
| *% active* | 0.10^1^ | 0.07 – 0.12 | 0.73^2^ | 0.59 – 0.91 | 0.83^2^ | 0.64 – 1.07 | 0.76^2^ | 0.58 – 1.01 | 3.13 | 0.0304 |  |
| *# inactive* | 36.50^1^ | 31.26 – 42.61 | 0.84^3^ | 0.71 – 0.99 | 0.90^3^ | 0.76 – 1.06 | 0.85^3^ | 0.70 – 1.02 | 1.84 | 0.1465 |  |
| *# inactive >15 min* | 4.79^1^ | 4.04 – 5.55 | 0.88^3^ | 0.09 – 1.68 | 0.68^3^ | -0.12 – 1.49 | 0.82^3^ | -0.10 – 1.73 | 1.92 | 0.1339 |  |

^1^ Estimated mean in reference night (night 1) for specified accelerometer output.

^2^ Estimated ratio of mean of specified night and mean of reference night for specified accelerometer output.

^3^ Estimated mean difference of specified night and reference night for specified accelerometer output.

**Supplementary Table S4:** **Full model results of nocturnal activity accelerometer outputs: Percentage of time spent active (*% active*).** Estimated parameter values (EP) and 95% confidence intervals (CI) of *% active* during the night (0:00-4:00 h) for night (after intake/adoption) and other factors that significantly explained *% active* variability. Conditional F-testing revealed F, DF’s and significance of factors in the model.

|  | | | *% active* | | | | | |
| --- | --- | --- | --- | --- | --- | --- | --- | --- |
| **Category** | | | Estimated | | Conditional F-test | | | |
|  |  |  | **EP** | **95% CI** | **F** | **NumDF** | **DenDF** | **Sign.** |
| Reference | Night 1, in the shelter, unknown history | | 0.23^1^ | 0.16 – 0.33 | 953.32 | 1 | 173 | <.0001 |
| Environment | Post adoption versus in the shelter | Night 1 | 0.43^2^ | 0.30 – 0.63 | 15.56 | 4 | 173 | <.0001 |
|  |  | Night 2 | 0.37^2^ | 0.26 – 0.53 |  |  |  |  |
|  |  | Night 3 | 0.56^2^ | 0.40 – 0.79 |  |  |  |  |
|  |  | Night 4 | 0.52^2^ | 0.36 – 0.76 |  |  |  |  |
| Kennel history | Had history versus unknown history | In the shelter | 0.56^3^ | 0.36 – 0.87 | 2.68 | 4 | 173 | 0.0336 |
|  |  | Post adoption | 0.93^3^ | 0.64 – 1.34 |  |  |  |  |
|  | No history versus unknown history | In the shelter | 1.15^3^ | 0.65 – 2.04 |  |  |  |  |
|  |  | Post adoption | 0.97^3^ | 0.59 – 1.59 |  |  |  |  |

^1^ Estimated mean in reference night, environment and kennel history.

^2^ Estimated ratio of mean of specified environment and mean of reference environment at the same night.

^3^ Estimated ratio of mean of specified kennel history and mean of reference kennel history in the same environment.

**Supplementary Table S5:** **Full model results of nocturnal activity accelerometer outputs: Number of inactive bouts (*# inactive*).** Estimated parameter values (EP) and 95% confidence intervals (CI) of *# inactive* during the night (0:00-4:00 h) for night (after intake/adoption) and other factors that significantly explained *# inactive* variability. Conditional F-testing revealed F, DF’s and significance of factors in the model.

|  | | | *# inactive* | | | | | |
| --- | --- | --- | --- | --- | --- | --- | --- | --- |
| **Category** | | | Estimated | | Conditional F-test | | | |
|  |  |  | **EP** | **95% CI** | **F** | **NumDF** | **DenDF** | **Sign.** |
| Reference | Night 1, in the shelter, relinquished, unknown history, short stay (<6 wks) | | 46.44^1^ | 36.01 – 59.89 | 5595.56 | 1 | 171 | <.0001 |
| Environment | Post adoption versus in the shelter | Night 1 | 0.59^2^ | 0.48 – 0.74 | 2.66 | 4 | 171 | 0.0347 |
|  |  | Night 2 | 0.59^2^ | 0.48 – 0.73 |  |  |  |  |
|  |  | Night 3 | 0.73^2^ | 0.59 – 0.89 |  |  |  |  |
|  |  | Night 4 | 0.62^2^ | 0.49 – 0.77 |  |  |  |  |
| Relinquishment type | Stray versus relinquished | In the shelter | 0.89^3^ | 0.69 – 1.14 | 7.73 | 2 | 171 | 0.0006 |
|  |  | Post adoption | 1.32^3^ | 1.04 – 1.69 |  |  |  |  |
| Kennel history | Had history versus unknown history | In the shelter | 0.71^4^ | 0.54 – 0.92 | 5.38 | 4 | 171 | 0.0004 |
|  |  | Post adoption | 1.28^4^ | 0.98 – 1.67 |  |  |  |  |
|  | No history versus unknown history | In the shelter | 1.04^4^ | 0.74 – 1.46 |  |  |  |  |
|  |  | Post adoption | 1.26^4^ | 0.88 – 1.79 |  |  |  |  |
| Time spent in shelter (in weeks) | Medium (6-12) versus short (<6) | | 1.25^5^ | 1.00 – 1.57 | 3.72 | 2 | 28 | 0.0369 |
|  | Long (>12) versus short (<6) | | 0.93^5^ | 0.72 – 1.21 |  |  |  |  |

^1^ Estimated mean in reference night, environment, relinquishment type, kennel history and time spent in the shelter.

^2^ Estimated ratio of mean of specified environment and mean of reference environment at the same night.

^3^ Estimated ratio of mean of specified relinquishment type and mean of reference relinquishment type in the same environment.

^4^ Estimated ratio of mean of specified kennel history and mean of reference kennel history in the same environment.

^5^ Estimated ratio of mean of specified time spent in shelter and mean of reference time spent in shelter.

**Supplementary Table S6:** **Full model results of nocturnal activity accelerometer outputs: Number of inactive bouts longer than 15 minutes (*# inactive >15 min*).** Estimated parameter values (EP) and 95% confidence intervals (CI) of *# inactive >15 min* during the night (0:00-4:00 h) for night (after intake/adoption) and other factors that significantly explained *# inactive >15 min* variability. Conditional F-testing revealed F, DF’s and significance of factors in the model.

|  | | | *# inactive >15 min* | | | | | |
| --- | --- | --- | --- | --- | --- | --- | --- | --- |
| **Category** | | | Estimated | | Conditional F-test | | | |
|  |  |  | **EP** | **95% CI** | **F** | **NumDF** | **DenDF** | **Sign.** |
| Reference | Night 1, in the shelter, relinquished, unknown history, short stay (<6 wks) | | 3.96^1^ | 2.86 – 5.07 | 805.15 | 1 | 171 | <.0001 |
| Environment | Post adoption versus in the shelter | Night 1 | 2.34^2^ | 1.12 – 3.57 | 4.98 | 4 | 171 | 0.0008 |
|  |  | Night 2 | 2.70^2^ | 1.50 – 3.90 |  |  |  |  |
|  |  | Night 3 | 1.33^2^ | 0.18 – 2.48 |  |  |  |  |
|  |  | Night 4 | 2.07^2^ | 0.80 – 3.34 |  |  |  |  |
| Relinquishment type | Stray versus relinquished | In the shelter | 0.50^3^ | -0.51 – 1.52 | 4.34 | 2 | 171 | 0.0146 |
|  |  | Post adoption | -1.11^3^ | -2.09 – -0.12 |  |  |  |  |
| Kennel history | Had history versus unknown history | In the shelter | 0.79^4^ | -0.28 – 1.86 | 2.66 | 4 | 171 | 0.0347 |
|  |  | Post adoption | -1.24^4^ | -2.30 – -0.17 |  |  |  |  |
|  | No history versus unknown history | In the shelter | -0.41^4^ | -1.78 – 0.95 |  |  |  |  |
|  |  | Post adoption | -0.23^4^ | -1.68 – 1.22 |  |  |  |  |
| Time spent in shelter (in weeks) | Medium (6-12) versus short (<6) | | -1.49^5^ | -2.33 – -0.65 | 7.09 | 2 | 28 | 0.0032 |
|  | Long (>12) versus short (<6) | | -0.48^5^ | -1.44 – 0.47 |  |  |  |  |

^1^ Estimated mean in reference night, environment, relinquishment type, kennel history and time spent in the shelter.

^2^ Estimated mean difference of specified environment and reference environment at the same night.

^3^ Estimated mean difference of specified relinquishment type and reference relinquishment type in the same environment.

^4^ Estimated mean difference of specified kennel history and reference kennel history in the same environment.

^5^ Estimated mean difference of specified time spent in shelter and reference time spent in shelter.

**Supplementary Table S7:** **Full model results of urinary cortisol:creatinine ratios (UCCR).** Estimated parameter values (EP) and 95% confidence intervals (CI) of *UCCR* during the night (0:00-4:00 h) for night (after intake/adoption) and other factors that significantly explained *UCCR* variability. Conditional F-testing revealed F, DF’s and significance of factors in the model.

|  | | | *UCCR* | | | | | |
| --- | --- | --- | --- | --- | --- | --- | --- | --- |
| **Category** | | | Estimated | | Conditional F-test | | | |
|  |  |  | **EP** | **95% CI** | **F** | **NumDF** | **DenDF** | **Sign.** |
| Reference | Day 1, in the shelter, <10 kg, no history | | 8.70^1^ | 5.85 – 12.96 | 290.14 | 1 | 218 | <.0001 |
| Environment | Post adoption versus in the shelter | Day 1 | 0.45^2^ | 0.32 – 0.64 | 20.50 | 6 | 218 | <.0001 |
|  |  | Day 2 | 0.35^2^ | 0.25 – 0.50 |  |  |  |  |
|  |  | Day 3 | 0.39^2^ | 0.28 – 0.55 |  |  |  |  |
|  |  | Day 7 | 0.35^2^ | 0.25 – 0.49 |  |  |  |  |
|  |  | Day 12 | 0.43^2^ | 0.31 – 0.62 |  |  |  |  |
|  |  | 6 weeks | 0.58^2^ | 0.39 – 0.86 |  |  |  |  |
| Weight class | 10-20 kg versus <10 kg | | 1.16^3^ | 0.78 – 1.71 | 6.43 | 3 | 27 | 0.0020 |
|  | >20-30 kg versus <10 kg | | 0.66^3^ | 0.44 – 0.97 |  |  |  |  |
|  | >30 kg versus <10 kg | | 0.49^3^ | 0.33 – 0.74 |  |  |  |  |
| Kennel history | Unknown history versus no history | In the shelter | 0.54^4^ | 0.36 – 0.80 | 3.00 | 4 | 218 | 0.0196 |
|  |  | Post adoption | 0.77^4^ | 0.54 – 1.10 |  |  |  |  |
|  | Had history versus no history | In the shelter | 0.97^4^ | 0.59 – 1.61 |  |  |  |  |
|  |  | Post adoption | 0.79^4^ | 0.47 – 1.32 |  |  |  |  |

^1^ Estimated mean in reference night, environment, weight class and kennel history.

^2^ Estimated ratio of mean of specified environment and mean of reference environment at the same night.

^3^ Estimated ratio of mean of specified weight class and mean of reference weight class.

^4^ Estimated ratio of mean of specified kennel history and mean of reference kennel history in the same environment.

**Supplementary Table S8:** **Full model results of UCCR after adoption.** Estimated parameter values (EP) and 95% confidence intervals (CI) of UCCR post adoption during the night (0:00-4:00 h) for night (after adoption) and other factors that significantly explained UCCR variability. Conditional F-testing revealed F, DF’s and significance of factors in the model.

|  | | *UCCR post adoption* | | | | | | |
| --- | --- | --- | --- | --- | --- | --- | --- | --- |
| **Category** | | Estimated | | Conditional F-test | | | | |
|  |  | **EP** | **95% CI** | **F** | **NumDF** | **DenDF** | **Sign.** |  |
| Reference | Night 1, <10 kg | 3.70^1^ | 2.76 – 4.96 | 152.34 | 1 | 74 | <0.0001 |  |
| Night | Night 2 versus night 1 | 0.81^2^ | 0.63 – 1.05 | 1.72 | 5 | 74 | 0.1407 |  |
|  | Night 3 versus night 1 | 0.84^2^ | 0.65 – 1.08 |  |  |  |  |  |
|  | Night 7 versus night 1 | 0.74^2^ | 0.58 – 0.96 |  |  |  |  |  |
|  | Night 12 versus night 1 | 0.75^2^ | 0.57 – 0.97 |  |  |  |  |  |
|  | 6 weeks versus night 1 | 0.73^2^ | 0.57 – 0.94 |  |  |  |  |  |
| Weight class | 10-20 kg versus <10 kg | 1.18^3^ | 0.76 – 1.83 | 4.40 | 3 | 22 | 0.0144 |  |
|  | >20-30 kg versus <10 kg | 0.66^3^ | 0.45 – 0.96 |  |  |  |  |  |
|  | >30 kg versus <10 kg | 0.59^3^ | 0.38 – 0.92 |  |  |  |  |  |

^1^ Estimated mean in reference night and weight class.

^2^ Estimated ratio of mean of specified night and mean of reference night.

^3^ Estimated ratio of mean of specified weight class and mean of reference weight class.

**Supplementary Table S9:** **Full model results of body weight proportional changes in the shelter.** Estimated parameter values (EP) and 95% confidence intervals (CI) of *proportional body weight* during the night (0:00-4:00 h) for night (after intake) and other factors that significantly explained *proportional body weight* variability. *Proportional body weight* at week 0 (intake) for all dogs is 1.000. Conditional F-testing revealed F, DF’s and significance of factors in the model.

|  | | | *Proportional body weight* | | | | | |
| --- | --- | --- | --- | --- | --- | --- | --- | --- |
| **Category** | | | Estimated | | Conditional F-test | | | |
|  |  |  | **EP** | **95% CI** | **F** | **NumDF** | **DenDF** | **Sign.** |
| Reference | Week 2, relinquished, <10 kg, medium-high food intake | | 0.927^1^ | 0.897 – 0.957 | 20419.47 | 1 | 27 | <.0001 |
| Week | Week 6 versus week 2 | | 0.039^2^ | -0.024 – 0.102 | 0.31 | 1 | 5 | 0.5995 |
| Weight class | 10-20 kg versus <10 kg | Week 2 | 0.047^3^ | -0.005 – 0.099 | 1.86 | 6 | 5 | 0.2559 |
|  |  | Week 6 | -0.037^3^ | -0.134 – 0.060 |  |  |  |  |
|  | >20-30 kg versus <10 kg | Week 2 | 0.040^3^ | -0.015 – 0.096 |  |  |  |  |
|  |  | Week 6 | -0.009^3^ | -0.086 – 0.069 |  |  |  |  |
|  | >30 kg versus <10 kg | Week 2 | 0.040^3^ | -0.016 – 0.096 |  |  |  |  |
|  |  | Week 6 | -0.040^3^ | -0.120 – 0.039 |  |  |  |  |
| Relinquishment type | Stray versus relinquished | | 0.030^4^ | -0.002 – 0.062 | 7.85 | 1 | 27 | 0.0093 |
| Food intake | Low versus medium-high | | -0.042^5^ | -0.080 – -0.003 | 7.98 | 1 | 27 | 0.0088 |

^1^ Estimated mean in reference week, weight class, relinquishment type and food intake.

^2^ Estimated mean difference of specified week and reference week.

^3^ Estimated mean difference of specified weight class and reference weight class at the same week.

^4^ Estimated mean difference of specified relinquishment type and reference relinquishment type.

^5^ Estimated mean difference of specified food intake and reference food intake.

**Supplementary Table S10:** **Full model results of body weight proportional changes after adoption.** Estimated parameter values (EP) and 95% confidence intervals (CI) of *proportional body weight* during the night (0:00-4:00 h) for night (after adoption) and other factors that significantly explained *proportional body weight* variability. *Proportional body weight* at week 0 (adoption) for all dogs is 1.000. Conditional F-testing revealed F, DF’s and significance of factors in the model.

|  | | *Proportional body weight* | | | | | | |
| --- | --- | --- | --- | --- | --- | --- | --- | --- |
| **Category** | | Estimated | | | Conditional F-test | | | |
|  |  | **EP** | **95% CI** | **F** | | **NumDF** | **DenDF** | **Sign.** |
| Reference | Week 2, <10 kg | 0.999^1^ | 0.972 – 1.026 | 14195.04 | | 1 | 22 | <.0001 |
| Week | Week 6 versus week 2 | 0.030^2^ | 0.011 – 0.048 | 11.67 | | 1 | 22 | 0.0025 |
| Weight class | 10-20 kg versus <10 kg | 0.001^3^ | -0.043 – 0.044 | 2.98 | | 3 | 20 | 0.0557 |
|  | >20-30 kg versus <10 kg | -0.020^3^ | -0.064 – 0.024 |  |  |  |  |  |
|  | >30 kg versus <10 kg | 0.081^3^ | 0.014 – 0.148 |  |  |  |  |  |

^1^ Estimated mean in reference week and weight class.

^2^ Estimated mean difference of specified week and reference week.

^3^ Estimated mean difference of specified weight class and reference weight class.
